# Diffusion adaptive filtering algorithm based on the Fair cost function

**DOI:** 10.1038/s41598-021-99330-9

**Published:** 2021-10-05

**Authors:** Sihai Guan, Qing Cheng, Yong Zhao, Bharat Biswal

**Affiliations:** 1 College of Electronic and Information, Southwest Minzu University, Chengdu, China; 2 Key Laboratory of Electronic and Information Engineering, State Ethnic Affairs Commission, Chengdu, China; 3 Sichuan Vocational College of Finance and Economics, Chengdu, China; 4 School of Mechanical and Power Engineering, Henan Polytechnic University, Jiaozuo, China; 5 The Clinical Hospital of Chengdu Brain Science Institute, MOE Key Laboratory for Neuroinformation, Center for Information in Medicine, School of Life Science and Technology, University of Electronic Science and Technology of China, Chengdu, China; 6 Department of Biomedical Engineering, New Jersey Institute of Technology (NJIT), Newark, NJ USA

**Keywords:** Information technology, Electrical and electronic engineering

## Abstract

To better perform distributed estimation, this paper, by combining the Fair cost function and adapt-then-combine scheme at all distributed network nodes, a novel diffusion adaptive estimation algorithm is proposed from an M-estimator perspective, which is called the diffusion Fair (DFair) adaptive filtering algorithm. The stability of the mean estimation error and the computational complexity of the DFair are theoretically analyzed. Compared with the robust diffusion LMS (RDLMS), diffusion Normalized Least Mean M-estimate (DNLMM), diffusion generalized correntropy logarithmic difference (DGCLD), and diffusion probabilistic least mean square (DPLMS) algorithms, the simulation experiment results show that the DFair algorithm is more robust to input signals and impulsive interference. In conclusion, Theoretical analysis and simulation results show that the DFair algorithm performs better when estimating an unknown linear system in the changeable impulsive interference environments.

## Introduction

Adaptive filter algorithms are often used in equalization, active noise control, echo cancellation, biomedical engineering, and many other fields^1–5^. Distributed adaptive signal processing is an extension of adaptive filters over graphs^6^. There are three widely-used distributed estimation cooperation strategies: incremental, consensus, and diffusion^3^. Besides, the asymmetry problem may lead to the unstable growth of consensus technology, and the asymmetry problem can be passed through the network. However, among them, the diffusion strategy can eliminate the asymmetry problem. So, the diffusion strategies were used frequently, and they include the adapt-then-combine (ATC) scheme^6^ and the combine-then-adapt (CTA) scheme^3,7^. The specific explanation is that in the CTA formulation of the diffusion strategy, the name combine-then-adapt is that the first step involves a combination step, while the second step involves an adaptation step; a similar implementation can be obtained by switching the order of the combination and adaptation steps. Moreover, Cattivelli and colleagues analyzed these two structures' performance for these two kinds of structures, showing that the ATC scheme outperforms the CTA scheme^6^. Then, the ATC scheme becomes a research focus in distributed adaptive filtering algorithms^6,8–15^. The Wiener filtering principle is the fundamental of the adaptive filtering algorithm^16^, based on the parameter mean square error (MSE) to construct an efficient cost function. Cost functions are used to learn parameters that explain the estimation well, and they define how costly our adaptive algorithm estimation mistakes are^17^. The cost function should penalize “large" estimation error and “very-large" estimation error almost equally. Also, if we want better estimation properties, then we have to give good computational complexity properties.

Roughly speaking, if a line connecting two points never intersects the cost function, the cost function is convex, and the convex cost function has only one global minimum value. Strictly convex cost functions have a unique global minimum value, and the sum of convex cost functions is also convex properties. Therefore, MSE has only one global minimum value. Furthermore, outliers are examples of data that are far from most other examples. Unfortunately, they happen more in reality than we hoped. Up to now, many types the cost functions have been used to design adaptive filtering algorithms, such as least mean absoult third^18^, least mean fourth^19^, entropy^20^, least-squares estimator^21^, absolute value estimator^22^, the Cauchy^23^, Geman–McClure^24^, Welsch^25,26^, and Huber function^27–30^. Specifically, the least-squares estimator is not robust because their influence function is not bounded^21^, and the absolute value estimator is not stable because the function of estimate error ($\left| {e\left( i \right)} \right|$) at *i*-th is not strictly convex^22^. Indeed, the second derivative at $e\left( i \right) = 0$ is unbounded, and an in determinant solution may result. The absolute value estimator reduces the influence of large errors, but they still have an influence because the influence function. Combine least-squares and absolute value estimator take both the absolute value estimators' advantage to reduce the influence of large errors and that of least-squares estimators to be convex^31^. They behave like least-squares for small $e\left( i \right)$ and like absolute value estimators for large $e\left( i \right)$, hence the name of this type of estimators. The least p-powers function represents a family of cost functions^32^. It is the least-square method when ${\text{p}} = 2$ and the absolute value estimator when ${\text{p}} = 1$. The smaller p, the smaller is the incidence of large estimation errors in the estimate ${\text{p}}$. It seems that p must be fairly moderate to provide a relatively reliable estimate, or in other words, an estimate that is hardly disturbed by outlying data. The selection of an optimal ${\text{p}}$ has been investigated, and for ${\text{p}}$ around 1.2, good estimated performance may be expected^33^. However, many difficulties are encountered when parameter ${\text{p}}$ is in the range of interest $1 < {\text{p}} < 2$, because zero residuals are troublesome. The other remaining functions have the same problem as the Cauchy function^23^. As can be seen from the influence function, the influence of large estimation errors only decreases linearly with their size. The Geman–McClure^24^ and Welsch^25,26^ functions try to further reduce the effect of large estimation errors. It seems complicated to select a cost function for general use without being somewhat arbitrary. The Huber cost function was often used^26–29^, but the Huber cost function is convex, differentiable, and robust to outliers. However, $\delta$ setting is not an easy task. Huber’s function is a parabola in the vicinity of zero, and increases linearly at a given level $\left| {e\left( i \right)} \right| > \delta$. This the estimator is very satisfactory and is rarely found that it is inferior to some other cost functions, so it is recommended in almost all situations. However, sometimes difficulties are encountered, which may be due to the discontinuous gradient value of the cost function due to its discontinuous second derivative. Tukey's loss is non-convex, non-differentiable^34^; it seems complicated to select a cost function for general use without being somewhat arbitrary. Following^35^, for the regression problems, the best choice is the Lp despite its theoretical no robustness: they are quasi-robust. However, it suffers from computational difficulties. However, the Fair function can yield nicely converging computational procedures. Compared to the quadratic cost function, the adaptive Fair function can track the statistical characteristics of the estimation error, which is conducive to improving the robustness of the proposed algorithm. In contrast, the Fair function is preferable since it is continuously derivable, while the Huber function is a piecewise function with unsmooth points.

Besides, system measurement interference will affect the error cost function, where impulse interference will significantly affect the estimation accuracy and most diffusion estimation algorithms on the network. Therefore, designing a robust distributed adaptive algorithm to deal with impulse interference of different intensities is necessary. Recently, Wen proposed the diffusion least mean p-power algorithm^36^, robust to the generalized Gaussian noise environment. Still, DLMP was proposed with a fixed power p-value, so p is the critical factor, which means the DLMP algorithm performance is highly susceptible to the p-value. Using minimization of L1-norm subject to a constraint on the estimate weight vectors, Ni and colleagues designed a diffusion sign subband adaptive filtering (DSSAF) algorithm^37^. Still, the computational complexity of the DSSAF algorithm is relatively large. By combining the diffusion least mean square (DLMS) algorithm^9^ and the sign operation to the estimated error at each iteration moment point, Ni and colleagues derived a diffusion sign-error LMS (DSELMS) algorithm^38^. The DSELMS algorithm architecture is simple and easy to implement, but the DSELMS algorithm has a significant drawback (i.e., the steady-state error is high)^39^. Besides, inspired by the least logarithmic absolute difference (LLAD) operation, Chen and colleagues designed the DLLAD algorithm^7^. Nevertheless, the robustness of this algorithm to the input signal and impulsive interference has not been performed. By combining the ATC strategy and the probabilistic LMS algorithm^8,40^, Guan and colleagues proposed a diffusion probabilistic least mean square (DPLMS) algorithm^41^. Based on the Huber objective function, a similar set of algorithms by Guan and colleagues^27^, Wei and colleagues^41^ have been proposed as the DNHuber DRVSSLMS algorithms, respectively. Besides, the computational algorithm complexity of the DRVSSLMS algorithm is high, which is not conducive to implementing practical engineering projects. Soheila and colleagues^29^ used the pseudo-Huber cost function instead of the square error to design the RDLMS algorithm based on the Huber cost function; however, the RDLMS algorithm was not designed for impulsive interference. By applying the modified Huber function together with an adaptive threshold, YU and colleagues proposed a novel diffusion normalized least mean M-estimate algorithm (DNLMM) against impulsive interference^30^ for impulsive interference. However, the basis of the DNLMM algorithm is still based on the Huber function. Huber’s function is a parabola in the vicinity of zero and increases linearly at a given level $\left| {e\left( i \right)} \right| > \delta$. And from time to time, difficulties are encountered, which may be due to the lack of stability in the gradient values of the cost function because of its discontinuous second derivative. Furthermore, the adaptive Fair function can track the statistical characteristics of the estimated error $e\left( i \right)$, which helps to improve the robustness of the proposed algorithm. In contrast, the Fair cost function is preferable since it is continuously derivable while the Huber cost function is a piecewise function with unsmooth points. So, we propose a robust distributed adaptive filtering algorithm by combining the Fair cost function and ATC scheme at all distributed network nodes in this paper, namely the DFair algorithm. The stability of the mean estimation error and the computational complexity are analyzed theoretically. Simulation experiment results indicate that the DFair algorithm is more robust to the input signal and impulsive interference than the RDLMS^29^, DNLMM^30^, DGCLD^42^, and DPLMS^41^ algorithms.

The remainder of this paper is organized as follows. The proposed DFair algorithm is proposed in detail in “Proposed the DFair algorithm” section. The DFair algorithm's statistical behavior, including the stability performance, computation complexity, and parameter ($\delta$) for the DFair algorithm, is studied in “Performance analysis” section. The simulation experiments are described in “Simulation results” section. Finally, the conclusion is provided in “Conclusion” section.

## Proposed the DFair algorithm

In this section, the DFair algorithm is developed. Firstly, an adaptive filtering algorithm based on the Fair cost function is proposed. Then we modify this adaptive filtering algorithm by extending at all network agents to develop the DFair algorithm.

### The adaptive filter algorithm based on the fair cost function

Let ${\mathbf{W}}\left( i \right)$ be the system estimated weight vector with length *M*, ${\mathbf{X}}\left( i \right)$ the input signal vector of the adaptive filter at iteration *i*, and the prediction error $e\left( i \right){ }$ between the desired signal $d\left( i \right)$ and the actual output $y\left( i \right)$ can be expressed by

1 $$ e\left( i \right) = d\left( i \right) - y\left( i \right) = {\mathbf{W}}^{{{\text{oT}}}} {\mathbf{X}}\left( i \right) + v\left( i \right) - {\mathbf{W}}^{{\text{T}}} \left( i \right){\mathbf{X}}\left( i \right) $$

where ${\mathbf{W}}^{{\text{o}}}$($M \times 1$) is the parameter of interest system, which needs to be estimated and $v\left( i \right)$ is the measurement interference.

Fair adaptive filter aims to minimize the Fair cost function defined as

2 $$ J\left( i \right) = \delta^{2} \left( {\frac{{\left| {e\left( i \right)} \right|}}{\delta } - log\left( {1 + \frac{{\left| {e\left( i \right)} \right|}}{\delta }} \right)} \right) $$

where $\delta > 0$ is the cut-off value.

According to the steepest descent method, the system estimated weight vector update of the Fair adaptive filter is

3 $$ {\mathbf{W}}\left( {i + 1} \right) = {\mathbf{W}}\left( i \right) + \mu \delta \left( {\frac{{\frac{{\left| {e\left( i \right)} \right|}}{\delta }}}{{1 + \frac{{\left| {e\left( i \right)} \right|}}{\delta }}}} \right)sgn\left( {e\left( i \right)} \right){\mathbf{X}}\left( i \right) = {\mathbf{W}}\left( i \right) + \mu \frac{\delta e\left( i \right)}{{\delta + \left| {e\left( i \right)} \right|}}{\mathbf{X}}\left( i \right) $$

where $sgn\left( \right)$ is the symbolic function, and $\mu$ is the step size.

### The adaptive diffusion filter based on the fair cost function

**Figure 1:**
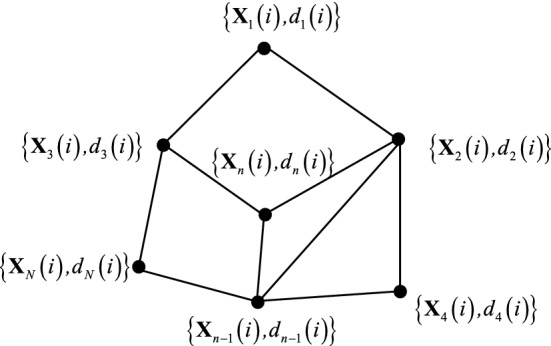
A network consisting of *N* agents^18,28,41^.

Our previous paper's research considers a network of *N* sensor nodes distributed over a geographic area (as Fig. 1)^18,28,41^. We assume an undirected graph so that if agent *n*-1 is a neighbor of agent *n*, then agent *n*-1 is also a neighbor of agent *n*. We assign a pair of nonnegative scaling weights to the edge connecting *n* and *n*-1. A network is connected if paths with nonzero scaling weights can be found linking any two distinct agents in both directions, either directly when they are neighbors or by passing through intermediate agents when they are not neighbors. In this way, information can flow in both directions between any two agents in the network. ${\mathbf{X}}_{n} \left( i \right)$ and $d_{n} \left( i \right)$ are the input signals and observation output signals at agent *n*, respectively.

An adaptive network equips the network's nodes with local learning rules or local adaptive filters. The available communication topology is then employed to efficiently implement a cooperation protocol among the nodes to exploit spatial and temporal information efficiently. Different learning rules allied with different cooperation protocols give rise to different adaptive networks. Based on Fig. 1, using the local cost function $J_{n}^{local} \left( {{\mathbf{W}}\left( i \right)} \right) = \delta^{2} \left( {\frac{{\left| {e_{n} \left( i \right)} \right|}}{\delta } - log\left( {1 + \frac{{\left| {e_{n} \left( i \right)} \right|}}{\delta }} \right)} \right)$, we seek the optimal linear estimator that minimizes the global cost function:

4 $$ J^{global} \left( {{\mathbf{W}}\left( i \right)} \right) = \mathop \sum \limits_{n} J_{n}^{local} \left( {{\mathbf{W}}\left( i \right)} \right) = \mathop \sum \limits_{n} \delta^{2} \left( {\frac{{\left| {e_{n} \left( i \right)} \right|}}{\delta } - log\left( {1 + \frac{{\left| {e_{n} \left( i \right)} \right|}}{\delta }} \right)} \right) $$

where at each time instant *i*, each sensor node ${ }n \in \left\{ {1,2, \cdots ,N} \right\}$ has access to some zero-mean random process ${ }\left\{ {d_{n} \left( i \right),{\mathbf{X}}_{n} \left( i \right)} \right\}$, $d_{n} \left( i \right)$ is a scalar and ${\mathbf{X}}_{n} \left( i \right)$ is a regression vector ($M \times 1$). Suppose these measurements output follows a standard model given by:

5 $$ d_{n} \left( i \right) = {\mathbf{X}}_{n}^{{\text{T}}} \left( i \right){\mathbf{W}}^{{\text{o}}} + v_{n} \left( i \right) $$

where ${\mathbf{W}}^{{\text{o }}}$($M \times 1$) is the unknown system parameter vector with length *M*, and $v_{n} \left( i \right)$ is the measurement interference with variance $\sigma_{v,n}^{2}$ and each node has a different value of $v_{n} \left( i \right)$.

In^9^, the DLMS algorithm is obtained by minimizing a linear combination of the local MSE:

6 $$ J_{n}^{local} \left( {{\mathbf{W}}\left( i \right)} \right) = \mathop \sum \limits_{{l \in N_{n} }} a_{l,n} {\text{E}}\left| {e_{l} \left( i \right)} \right| = \mathop \sum \limits_{{l \in N_{n} }} a_{l,n} {\text{E}}\left| {d_{l} \left( i \right) - {\mathbf{X}}_{l}^{{\text{T}}} \left( i \right){\mathbf{W}}\left( i \right)} \right| $$

where the set of nodes that are connected to *n* (including *n* itself) is denoted by $$N_{n}$$ and is called the neighborhood of nodes *n*. The weighting coefficients $$a_{l,n}$$ are real and satisfy $$ \sum\nolimits_{{l \in N_{n} }} {a_{l,n} } = 1$$. $$a_{l,n} $$ forms a nonnegative combination matrix ***A***.

The DLMS algorithm obtains the estimation via two steps, adaptation and combination. According to the order of these two steps, the updating equation of the DLMS algorithm can be expressed as

7 $$ \left\{ {\begin{array}{*{20}c} {{\mathbf{\varphi }}_{n} \left( i \right) = {\mathbf{W}}_{n} \left( {i - 1} \right) + \mu_{n} {\mathbf{X}}_{n} \left( i \right)e_{n} \left( i \right)} \\ {{\mathbf{W}}_{n} \left( i \right) = \mathop \sum \limits_{{l \in N_{n} }} a_{l,n} {\mathbf{\varphi }}_{l} \left( i \right)} \\ \end{array} } \right. $$

where $\mu_{n}$ is the step size (learning rate), and ${ }{\mathbf{\varphi }}_{n} \left( i \right)$ is the local estimates at node *n*.

Based on Eq. (7), the derivative of the local instantaneous approximations for ${\mathbf{W}}_{n} \left( i \right)$ can be formulated as:

8 $$ \nabla J_{n}^{local} \left( {{\mathbf{W}}_{n} \left( i \right)} \right) = \frac{\partial }{{\partial {\mathbf{W}}\left( i \right)}}\left( {J_{n}^{local} \left( {{\mathbf{W}}_{n} \left( i \right)} \right)} \right) = - \frac{{\delta e_{n} \left( i \right)}}{{\delta + \left| {e_{n} \left( i \right)} \right|}}{\mathbf{X}}_{n} \left( i \right) $$

As shown in Fig. 1 for the framework of the ATC diffusion strategy, the diffusion algorithms first update the local intermediate estimate by the steepest descent method. Then, each node combines the local intermediate estimates from its neighbors. The steps of the diffusion strategy for distributed estimation are as follows: (1) adaptation step, utilizing the stochastic gradient descent method, the intermediate estimate of node k for the parameter update is derived as:

9 $$ {\mathbf{\varphi }}_{n} \left( i \right) = {\mathbf{W}}_{n} \left( {i - 1} \right) - \mu_{n} \nabla J_{n}^{local} \left( {{\mathbf{W}}_{n} \left( i \right)} \right) = {\mathbf{W}}_{n} \left( {i - 1} \right) + \mu_{n} \frac{{\delta e_{n} \left( i \right)}}{{\delta + \left| {e_{n} \left( i \right)} \right|}}{\mathbf{X}}_{n} \left( i \right) $$

where $\mu_{n}$ is a learning step-size and ${\mathbf{W}}_{n} \left( i \right)$ is the estimate of ${\mathbf{W}}^{{\text{o}}}$ for node $n$ at the time instant $i$.

(2) combination step, in this step, the node receives all intermediate estimates from its neighbors as follows:

10 $$ {\mathbf{W}}_{n} \left( i \right) = \mathop \sum \limits_{{l \in N_{n} }} a_{l,n} {\mathbf{\varphi }}_{l} \left( i \right) $$

For simplicity, a summary of the DFair algorithm procedure based on the analysis presented above is given in Table 1.

**Table 1:**
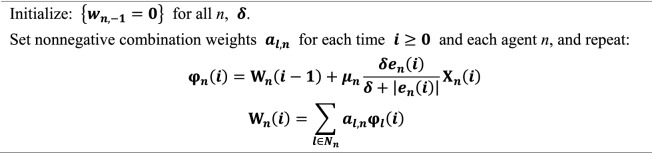
The DFair algorithm summary.

## Performance analysis

The DFair adaptive filtering algorithms, including mean behavior and computational complexity, will be discussed in this subsection. Firstly, to facilitate analysis and expression, we define some equations at agent *n* and time *i*, ${\hat{\mathbf{W}}}_{n} \left( i \right) = {\mathbf{W}}^{{\text{o}}} - {\mathbf{W}}_{n} \left( i \right)$, ${\hat{\mathbf{\varphi }}}_{n} \left( i \right) = {\mathbf{W}}^{{\text{o}}} - {\mathbf{\varphi }}_{n} \left( i \right)$, which are then collected to form the system weight error vector and intermediate system weight error vector, i.e., ${\mathbf{W}}\left( i \right) = {\text{col}}\left\{ {{\mathbf{W}}_{1} \left( i \right),{\mathbf{W}}_{2} \left( i \right), \ldots ,{\mathbf{W}}_{N} \left( i \right)} \right\}$, ${\mathbf{\varphi }}\left( i \right) = {\text{col}}\left\{ {{\mathbf{\varphi }}_{1} \left( i \right),{\mathbf{\varphi }}_{2} \left( i \right), \ldots ,{\mathbf{\varphi }}_{N} \left( i \right)} \right\}$, ${\hat{\mathbf{W}}}\left( i \right) = {\text{col}}\left\{ {{\hat{\mathbf{W}}}_{1} \left( i \right),{\hat{\mathbf{W}}}_{2} \left( i \right), \ldots ,{\hat{\mathbf{W}}}_{N} \left( i \right)} \right\}$, ${\hat{\mathbf{\varphi }}}\left( i \right) = {\text{col}}\left\{ {{\hat{\mathbf{\varphi }}}_{1} \left( i \right),{\hat{\mathbf{\varphi }}}_{2} \left( i \right), \ldots ,{\hat{\mathbf{\varphi }}}_{N} \left( i \right)} \right\}$, ${{\varvec{\upmu}}}\left( i \right) = {\text{diag}}\left\{ {\mu_{1} \frac{\delta }{{\delta + \left| {e_{1} \left( i \right)} \right|}},\mu_{2} \frac{\delta }{{\delta + \left| {e_{2} \left( i \right)} \right|}}, \ldots ,\mu_{N} \frac{\delta }{{\delta + \left| {e_{N} \left( i \right)} \right|}}} \right\}$, and ${\mathbf{e}}\left( i \right) = {\text{col}}\left\{ {e_{1} \left( i \right),e_{2} \left( i \right), \cdots ,e_{N} \left( i \right)} \right\}$.

### Mean weight error vector behavior

To facilitate performance analysis, we make the following assumptions:

#### **Assumption 1**

All measurement interferences are independent of any other signals.

#### **Assumption 2**

${\mathbf{X}}\left( i \right)$ is zero-mean Gaussian, temporally white, and spatially independent with ${\mathbf{R}}_{xx,n} = {\text{E}}\left[ {{\mathbf{X}}_{{\varvec{n}}} \left( i \right){\mathbf{X}}_{{\varvec{n}}}^{{\text{T}}} \left( i \right)} \right]$.

#### **Assumption 3**

The regression vector ${\mathbf{X}}_{n} \left( i \right)$ is independent of ${\hat{\mathbf{W}}}_{k} \left( j \right)$ for all k and j < i.

The DFair algorithm will be obtained. Equations (9) and (10) can be written as

11 $$ {\hat{\mathbf{\varphi }}}\left( i \right) = {\hat{\mathbf{W}}}\left( {i - 1} \right) - {\varvec{S}}_{{{\varvec{\upmu}}}} {\mathbf{S}}_{{{\mathbf{S}}_{{\mathbf{e}}} }} \left( i \right){\mathbf{X}}\left( i \right) $$

12 $$ {\hat{\mathbf{W}}}\left( i \right) = {\mathbf{C}}^{{\text{T}}} {\hat{\mathbf{\varphi }}}\left( i \right) $$

where ${ }{\mathbf{C}} = {\mathbf{C}} \otimes {\mathbf{I}}$, ${\varvec{S}}_{{{\varvec{\upmu}}}} = {{\varvec{\upmu}}} \otimes {\mathbf{I}}$, ${\mathbf{S}}_{{{\mathbf{S}}_{{\mathbf{e}}} }} \left( i \right) = {\mathbf{S}}_{{\mathbf{e}}} \left( i \right) \otimes {\mathbf{I}}$, ${\mathbf{X}}\left( i \right) = {\text{col}}\left\{ {{\mathbf{X}}_{1} \left( i \right),{\mathbf{X}}_{2} \left( i \right), \ldots ,{\mathbf{X}}_{N} \left( i \right)} \right\}$**,** ${\mathbf{S}}_{{\mathbf{e}}} \left( i \right) = {\text{diag}}\left\{ {{\mathbf{e}}\left( i \right)} \right\},$ and ${ } \otimes$ denotes the Kronecker product operation.

Taking the expectation of Eqs. (11) and (12),

13 $$ {\text{E}}\left[ {{\hat{\mathbf{W}}}\left( i \right)} \right] = {\mathbf{C}}^{{\text{T}}} {\mathbf{E}}\left[ {{\hat{\mathbf{W}}}\left( {i - 1} \right)} \right] - {\mathbf{C}}^{{\text{T}}} {\varvec{S}}_{{{\varvec{\upmu}}}} {\mathbf{E}}\left[ {{\mathbf{S}}_{{{\mathbf{S}}_{{\mathbf{e}}} }} \left( i \right){\mathbf{X}}\left( i \right)} \right] $$

Denote the measurement interference vector by ${\mathbf{V}}\left( i \right) = {\text{col}}\left\{ {v_{1} \left( i \right),v_{2} \left( i \right), \ldots ,v_{N} \left( i \right)} \right\}$, ${\mathbf{g}}\left( i \right) = {\text{col}}\left\{ {g_{1} \left( i \right),g_{2} \left( i \right), \ldots ,g_{N} \left( i \right)} \right\}$, ${\mathbf{Im}}\left( i \right) = {\text{col}}\left\{ {Im_{1} \left( i \right),Im_{2} \left( i \right), \ldots ,Im_{N} \left( i \right)} \right\}$, ${\mathbf{S}}_{{\mathbf{g}}} \left( i \right) = {\text{diag}}\left\{ {{\mathbf{g}}\left( i \right)} \right\}$, ${\mathbf{S}}_{{{\mathbf{Im}}}} \left( i \right) = {\text{diag}}\left\{ {{\mathbf{Im}}\left( i \right)} \right\}$, ${\mathbf{S}}_{{\varvec{X}}} \left( i \right) = {\text{diag}}\left\{ {{\mathbf{X}}_{1} \left( i \right),{\mathbf{X}}_{2} \left( i \right), \ldots ,{\mathbf{X}}_{N} \left( i \right)} \right\}$. So, from Eq. (1) we have ${\mathbf{e}}\left( i \right) = {\mathbf{S}}_{{\varvec{X}}}^{{\text{T}}} \left( i \right){\hat{\mathbf{W}}}\left( {i - 1} \right) + {\mathbf{V}}\left( i \right) = {\mathbf{e}}_{{\varvec{o}}} \left( i \right) + {\mathbf{V}}\left( i \right)$. Then, let $$\left\{ {\begin{array}{*{20}c} {{\mathbf{e}}_{{\varvec{g}}} \left( i \right) = {\mathbf{e}}_{{\varvec{o}}} \left( i \right) + g\left( i \right)} \\ {{\mathbf{e}}_{{{\varvec{Im}}\left( i \right)}} = {\mathbf{e}}_{{\varvec{o}}} \left( i \right) + Im\left( i \right)} \\ \end{array} } \right.$$, $$\left\{ {\begin{array}{*{20}c} {{\varvec{S}}_{{{\mathbf{S}}_{{\varvec{g}}} }} \left( i \right) = {\mathbf{S}}_{{\varvec{g}}} \left( i \right) \otimes I} \\ {{\varvec{S}}_{{{\mathbf{S}}_{{{\varvec{Im}}}} }} \left( i \right) = {\mathbf{S}}_{{{\varvec{Im}}}} \left( i \right) \otimes I} \\ \end{array} } \right.$$.

So,

14 $$ {\text{E}}\left[ {{\hat{\mathbf{W}}}\left( i \right)} \right] = {\mathbf{C}}^{{\text{T}}} \left[ {{\varvec{I}}_{{{\varvec{NM}}}} - {\varvec{S}}_{{{\varvec{\upmu}}}} {\text{diag}}\left\{ {{\mathbf{R}}_{xx,1} ,{\mathbf{R}}_{xx,2} , \ldots ,{\mathbf{R}}_{xx,N} } \right\}} \right]{\mathbf{E}}\left[ {{\hat{\mathbf{W}}}\left( {i - 1} \right)} \right] $$

From Eq. (14), one can see that the asymptotic unbiasedness of the DFair algorithm can be guaranteed if the matrix ${\mathbf{C}}^{{\text{T}}} \left[ {{\varvec{I}}_{{{\varvec{NM}}}} - {\varvec{S}}_{{{\varvec{\upmu}}}} {\text{diag}}\left\{ {{\mathbf{R}}_{xx,1} ,{\mathbf{R}}_{xx,2} , \ldots ,{\mathbf{R}}_{xx,N} } \right\}} \right]$ is stable. The matrix $\left[ {{\varvec{I}}_{{{\varvec{NM}}}} - {\varvec{S}}_{{{\varvec{\upmu}}}} {\text{diag}}\left\{ {{\mathbf{R}}_{xx,1} ,{\mathbf{R}}_{xx,2} , \ldots ,{\mathbf{R}}_{xx,N} } \right\}} \right]$ is a block-diagonal matrix and it can be easily verified that it is stable if its block-diagonal entries $\left[ {{\varvec{I}} - \mu_{n} {\mathbf{R}}_{xx,n} } \right]$ is stable. So, the condition for stability of the mean weight error vector (as Eq. (15)) is given by

15 $$ 0 < \mu_{n} < \frac{2}{{\rho_{max} \left( {{\mathbf{R}}_{xx,n} } \right)}} $$

where $\rho_{max}$ denotes the maximal eigenvalue of ${\mathbf{R}}_{xx,n}$. So, based on Eqs. (13) and (15), we obtain ${\text{ E}}\left[ {{\hat{\mathbf{W}}}\left( \infty \right)} \right] = 0$.

### Parameters δ for the proposed algorithm

The choice of $\delta$ in Eq. (8) plays a vital role in the performance of the DFair algorithm. When $\left| {e_{n} \left( i \right)} \right|/\delta \to 0$, $\hat{\mu }_{n} \left( i \right) = 0$ for each time ${ }i \ge 0{ }$ and each agent *n*. When $\left| {e_{n} \left( i \right)} \right|/\delta \to \infty$, $\hat{\mu }_{n} \left( i \right) = \mu_{n}$ for each time ${ }i \ge 0{ }$ and each agent *n*. So, when large $\hat{\mu }_{n} \left( i \right) = \mu_{n}$ lead to a large MSD and even cause loss of convergence, while a small $\hat{\mu }_{n} \left( i \right) = 0$ degrade the tracking speed of the DFair algorithm, which means that a large step-size responds quickly to plant changes during the initial convergence, and then a tiny step-size is used as the algorithm approaches its steady state. In other words, the DFair algorithm has been presented to obtain a fast convergence rate and a small steady-state error. Therefore, it is necessary to discuss the value of $\delta$ under the different intensities of impulsive interference. We set six experiment groups in a system identification application to choose the optimum cut-off value δ under different input signals, impulsive interference, and different network structures. Another method that can get the optimum cut-off value $\delta$ based on the theory derivation method for different input signals, various impulsive interferences, and various network structures. For the theory derivation method, although the optimal parameters $\delta { }$ of the proposed two algorithms are obtained based on minimizing the mean-square deviation (MSD) at the current time. The specific derivation methods are similar so that readers can refer to our previous published paper for details^43^. However, the optimal parameters $\delta$ must be time-varying with MSD, which increases the complexity of the algorithm. Therefore, for the sake of simplicity and this paper mainly discusses the design of a novel cost function structure, iterative formulas will increase the computational complexity. So, in this paper, find the approximate optimal parameter value $\delta$ of the proposed diffusion adaptive filtering algorithm by designing multiple sets of simulation experiments in different situations. Several experiments were performed in a system identification application in the presence of impulsive interference and Gaussian noise. Gaussian noise is a white Gaussian random process with zero mean and variance equal to 0.01. Impulsive interference is a Bernoulli–Gaussian distribution^18^ that was added to the unknown system output also. The Bernoulli–Gaussian impulsive interference, $v\left( i \right) = f\left( i \right)g\left( i \right)$ is a product of a Bernoulli process ${ }g\left( i \right){ }$ and a Gaussian process $f\left( i \right)$, where ${ }f\left( i \right){ }$ is a white Gaussian random process with zero mean and variance $\sigma_{f}^{2}$, and $g\left( i \right) = \left\{ {0,{ }1} \right\}{ }$ is a Bernoulli process with the probabilities $p\left( 1 \right) = {\text{Pr}}$ and $p\left( 0 \right) = 1 - {\text{Pr}}$.

**Figure 2:**
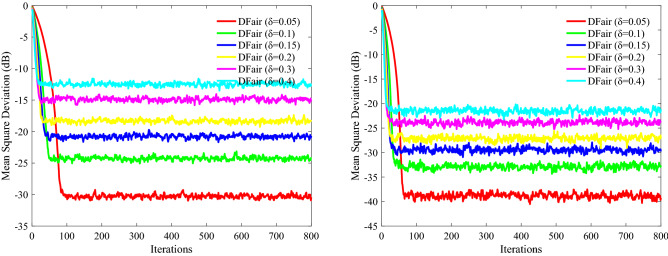
MSD curve with different $\delta$ of the DFair algorithm (*μ* = 0.4) when network topology and neighbors to be decided by probability (probability = 0.2): (Left) ${\mathbf{R}}_{xx,n} = \sigma_{x,n}^{2} {\mathbf{I}}_{M}$, Pr = 0.6, and $\sigma_{f}^{2} = 0.5$. (Right) ${\mathbf{R}}_{xx,n} = \sigma_{x,n}^{2} \left( i \right){\mathbf{I}}_{M} ,i = 1,2,3, \ldots ,M$, Pr = 0.2, and $\sigma_{f}^{2} = 0.5$.

**Figure 3:**
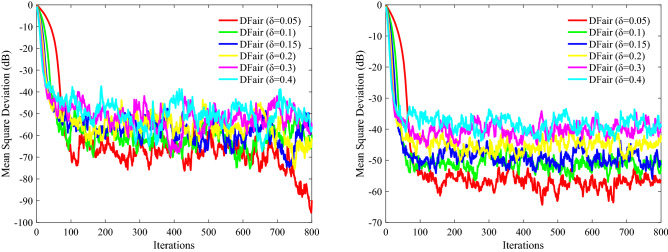
MSD curve with different $\delta$ of the DFair algorithm (*μ* = 0.4) when network topology and neighbors to be decided by closeness in the distance (radius = 0.3): (Left) ${\mathbf{R}}_{xx,n}$ is a diagonal matrix with possibly different diagonal entries chosen randomly, Pr = 0.4, and $\sigma_{f}^{2} = 0.5$. (Right) ${\mathbf{R}}_{xx,n} = \sigma_{x,n}^{2} \left( i \right){\mathbf{I}}_{M} ,i = 1,2,3, \ldots ,M$, Pr = 0, and $\sigma_{f}^{2} = 0.5$.

In this part, nodes of network topology are set as *N* = 20, and the input regresses ${\mathbf{X}}_{n} \left( i \right)$ of this distributed network are assumed to be spatiotemporally independent zero-mean white Gaussian distributed with different covariance matrixes ${\mathbf{R}}_{xx,n}$. The impulsive interference is also assumed to be spatially and temporally independent distributed with power $\user2{ }\sigma_{f}^{2}$. For the adaptation and combination weights, we apply the uniform rule (i.e., $a_{l,n} = 1/N_{n}$, where the set of nodes that are connected to *n* is denoted by $N_{n}$). We evaluate the relative efficiency of different $\delta$ estimators based on their MSD to evaluate the performance of DFair, where ${\text{ MSD}}\left( i \right) = \frac{1}{N}\mathop \sum \limits_{n = 1}^{N} {\text{E}}\left[ {\left| {{\mathbf{W}}_{o} - {\mathbf{W}}_{n} \left( i \right)} \right|^{2} } \right]$^28,41^. Also, the independent Monte Carlo number is 10, and each run has 800 iteration numbers. The different probability density of impulsive interference is considered 0%, 20%, 40%, 60%. Figures 2, 3(Left), and 4(Left) considering the convergence rate and the steady-state estimated error, we know the DFair algorithm is robust for the different probability density of interference when $\delta = 0.1$.

**Figure 4:**
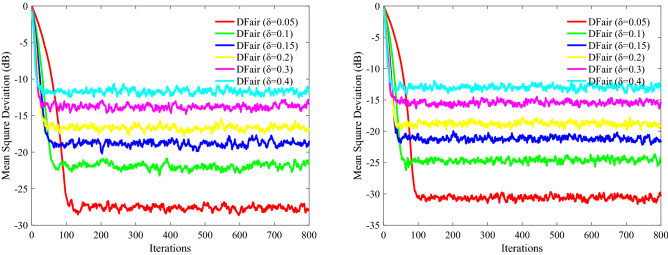
MSD curve with different $\delta$ of the DFair algorithm (*μ* = 0.4) when network topology and neighbors to be decided by closeness in the distance (radius = 0.3): (Left) ${\mathbf{R}}_{xx,n}$ is a diagonal matrix with possibly different diagonal entries chosen randomly, Pr = 0.4, and $\sigma_{f}^{2} = 0.2$. (Right) ${\mathbf{R}}_{xx,n} = \sigma_{x,n}^{2} \left( i \right){\mathbf{I}}_{M} ,i = 1,2,3, \ldots ,M$, Pr = 0.4, and $\sigma_{f}^{2} = 0.4$.

### Computational complexity

The adaptive filtering algorithm's computational complexity is the number of arithmetic operations per iteration of the weight vector or coefficient vector. That is the number of multiplications, additions, and et.al. The multiplication operation's time-consuming operation is far greater than the addition operation's time-consuming operation, so the multiplication operation occupies a large proportion of the adaptive filtering algorithm's computational complexity. Therefore, computational complexity is an important property that affects the performance of the adaptive filtering algorithm. According to the above description, the DFair algorithm can be regarded as the structure of the variable step size DLMS algorithm with the following characteristics: $\hat{\mu }_{n} \left( i \right) = \mu_{n} \frac{{\delta e_{n} \left( i \right)}}{{\delta + \left| {e_{n} \left( i \right)} \right|}}$ (from Table 1). The DFair algorithm has only two more multiplication operations than the DLMS algorithm. But for DPLMS, there are need more multiplication operations when $\mu_{n} \left( i \right) = \mu_{n} \alpha_{n} \left( i \right)$ in Eq. (16)^41^ will be computed. Also, for the RDLMS algorithm, there have more multiplication operations when $\hat{\mu }_{n} \left( i \right) = \mu_{n} \frac{{b_{n} e_{n} \left( i \right)}}{{\sqrt {1 + \left( {e_{n} \left( i \right)/\delta } \right)^{2} } }}$ in Eq. (17)^29^ will be run. In addition, for the DNLMM algorithm, $\hat{\mu }_{n} \left( i \right) = \mu_{n} \frac{{e_{n} \left( i \right)}}{{\sqrt {1 + {\mathbf{X}}_{{\varvec{n}}} \left( i \right)_{2}^{2} } }}$ in Eq. (13a) and Eq. (15)^30^ will be consider. Furthermore, $\hat{\mu }_{n} \left( i \right) = \mu_{n} \left( {1 + \frac{{e_{n} \left( i \right)}}{{\delta \left( {1 + \delta \left[ {exp\left( { - \lambda e_{k} \left( {i - 1} \right)^{\alpha } } \right)} \right]} \right)}}} \right)$ in Table 1 and Eq. (15)^42^ will add more multiplication operations. So, the computational complexity of the DFair algorithm smaller than RDLMS^29^, DNLMM^30^, DGCLD^42^, and DPLMS^41^ algorithms.

## Simulation results

In this paper, we focus on the distributed adaptive filtering algorithm and compare the DFair algorithm with the RDLMS^29^, DNLMM^30^, DGCLD^42^, and DPLMS^41^ algorithms in linear system identification under different types of input signal and impulsive interference. In this part, to demonstrate the robustness performance of the proposed DFair algorithm in the presence of different intensity levels of impulsive interference and input signal, we set several group simulation experiments with different impulsive interference and different input signal types. For this unknown linear system, we set *M* = 8, and the parameters vector was selected randomly. Each distributed network topology consists of $N = 20$ nodes. An impulsive interference with a Bernoulli–Gaussian distribution^18^ was added to the system output (as described in “Computational complexity” section). Besides, we set the impulsive interference as spatiotemporally independent. For the adaptation weights in the adaptation step and combination weights in the combination step, we apply the uniform rule i.e. $a_{l,n} = 1/N_{n}$. We use the network MSD to evaluate the performance of diffusion algorithms^28,41^.

### Simulation experiment 1

**Figure 5:**
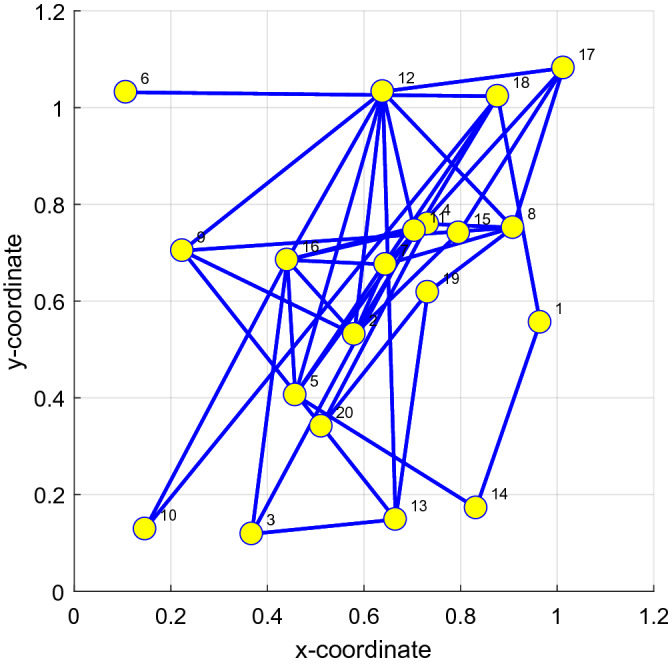
Random network topology is to be decided by probability.

**Figure 6:**
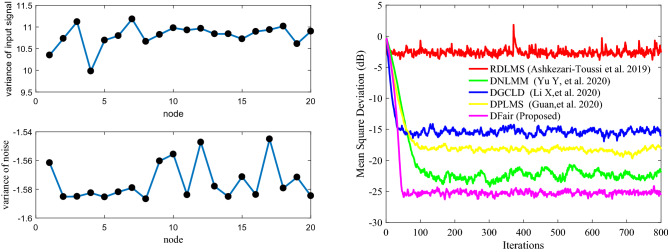
(Left_top) the input signals $\left\{ {{\mathbf{X}}_{n} \left( i \right)} \right\}$ variances of at each network node with ${ }{\mathbf{R}}_{xx,n}$ is a diagonal matrix with possibly different diagonal entries chosen randomly, (Left_bottom) the measurement interference variances $\varepsilon_{n} \left( i \right)$ at each network node; (Right) Transient network MSD (dB) iteration curves of the RDLMS, DNLMM, DGCLD, DPLMS, and DFair algorithms.

**Figure 7:**
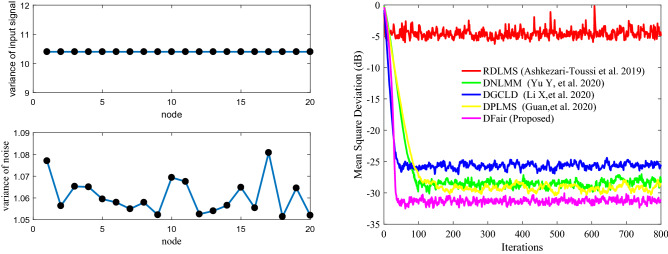
(Left_top) the input signals $\left\{ {{\mathbf{X}}_{n} \left( i \right)} \right\}$ variances of at each network node with ${\mathbf{R}}_{xx,n} = \sigma_{x,n}^{2} {\mathbf{I}}_{M}$, (Left_bottom) the measurement interference variances $\varepsilon_{n} \left( i \right)$ at each network node; (Right) Transient network MSD (dB) iteration curves of the RDLMS, DNLMM, DGCLD, DPLMS, and DFair algorithms.

**Figure 8:**
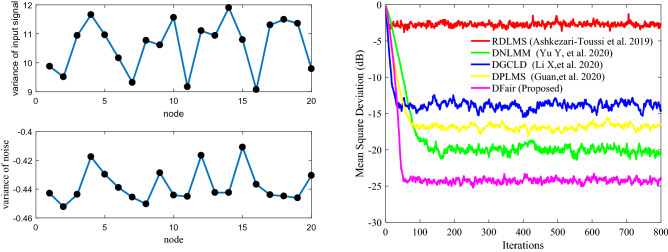
(Left_top) the input signals $\left\{ {{\mathbf{X}}_{n} \left( i \right)} \right\}$ variances of at each network node with ${\mathbf{R}}_{xx,n} = \sigma_{x,n}^{2} \left( t \right){\mathbf{I}}_{M} ,t = 1,2,3, \ldots ,M$, (Left_bottom) the measurement interference variances $\varepsilon_{n} \left( i \right)$ at each network node; (Right) Transient network MSD (dB) iteration curves of the RDLMS, DNLMM, DGCLD, DPLMS, and DFair algorithms.

Illustrating our proposed DFair algorithm is more robust to the input signal than the RDLMS, DNLMM, DGCLD, and DPLMS algorithms. In this experiment, there have the same network topology and the same impulsive interference. If any two network topology nodes are declared neighbors, connect probability greater than or equal to 0.2, the network topology is shown in Fig. 5. The MSD iteration curves for RDLMS ($\mu$ equal to 0.4), DNLMM ($\mu$ equal to 0.4), DGCLD ($\mu$ equal to 0.4), DPLMS, and DFair ($\mu$ equal to 0.4) algorithms in Figs. 6, 7, and 8 are different types of the input signal when the measurement interference in an unknown linear system is impulsive interference with Pr = 0.4, ${ }\sigma_{f}^{2} = 0.09$ and the cut-value $\delta = 0.1$ for the DFair algorithm. Besides, the independent Monte Carlo number is 10, and each run has 800 iteration numbers.

Figures 6, 7, and 8 show that although different input signals are used, the DFair algorithm still has a faster convergence rate and lowest steady-state estimated error than the RDLMS, DNLMM, DGCLD, and DPLMS algorithms. Besides, the DFair algorithm is more robust to the input signal. In a word, from *Simulation experiment 1*, we can get the DFair algorithm superior to the RDLMS, DNLMM, DGCLD, and DPLMS algorithms with different input signals, impulsive interference when using the same distributed network topology.

### Simulation experiment 2

**Figure 9:**
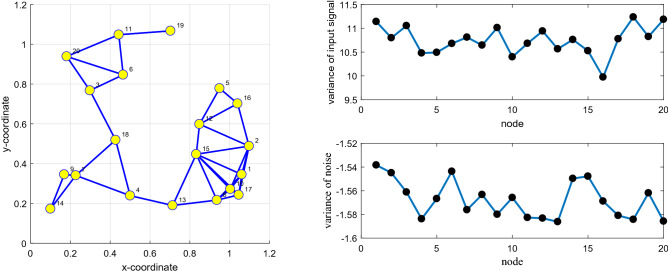
(Left) Random network topology to be decided by a certain radius; (Right_top) the input signals $\left\{ {{\mathbf{X}}_{n} \left( i \right)} \right\}$ variances of at each network node with ${\mathbf{R}}_{xx,n}$ is a diagonal matrix with possibly different diagonal entries chosen randomly, (Right_bottom) the measurement interference variances $\varepsilon_{n} \left( t \right)$ at each network node.

**Figure 10:**
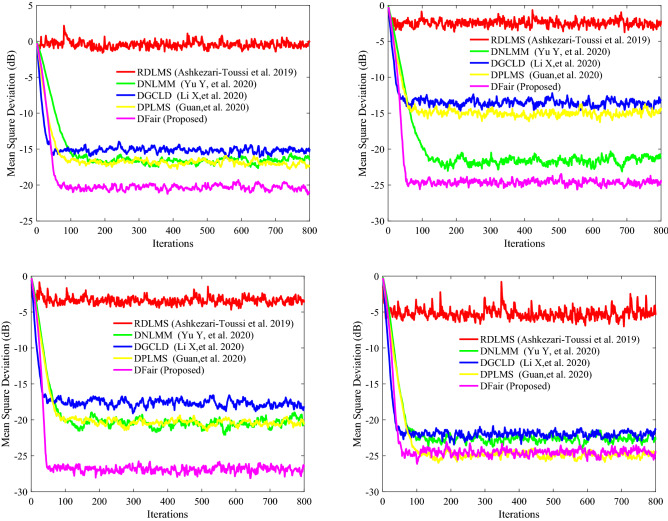
Transient network MSD (dB) iteration curves of the RDLMS, DNLMM, DGCLD, DPLMS, and DFair algorithms. (Up_Left) with, $\sigma_{f}^{2} = 0.04$, (Up_right) with $\sigma_{f}^{2} = 0.06$, and (Down_left) with $\sigma_{f}^{2} = 0.08$, and (Down_Right) with $\sigma_{f}^{2} = 0.1$.

Illustrating the DFair algorithm is more robust to $\sigma_{f}^{2} { }$ for impulsive interference with a constant ${\text{Pr}}$ and faster convergence rate and lower steady-state estimated error than the RDLMS, DNLMM, DGCLD, and DPLMS algorithms. This experiment has the same network topology: the same Pr of impulsive interference and the same input signal. If any two nodes in network topology are declared neighbors, a certain radius for each node is larger than or equal to 0.3, and the network topology is shown in Fig. 9(Left). The MSD iteration curves for RDLMS ($\mu$ equal to 0.3), DNLMM ($\mu$ equal to 0.3), DGCLD ($\mu$ equal to 0.3), DPLMS, and DFair ($\mu$ equal to 0.3) algorithms in Fig. 10 with Pr = 0.4 and the cut-value $\delta = 0.1$ for the DFair algorithm. Also, the independent Monte Carlo number is 10, and each run has 800 iteration numbers.

In this experiment, we want to show that the DFair algorithm is more robust to different the probability density of impulsive interference, so we set four sub-experiments with different density $\sigma_{f}^{2}$ of impulsive interference, the same Pr of impulsive interference, the same input signal, and the same distributed network topology. From Fig. 10, we can find that although different probability density of impulsive interference is considered, the DFair algorithms have a slightly faster rate than the RDLMS, DNLMM, DGCLD, and DPLMS algorithms. The DFair algorithm still has a minor steady-state error than the RDLMS, DNLMM, DGCLD, and DPLMS algorithms. In a word, from *Simulation experiment 2*, we can observe that the DFair algorithm is more robust to impulsive interference than the RDLMS, DNLMM, DGCLD, and DPLMS algorithms.

### Simulation experiment 3

**Figure 11:**
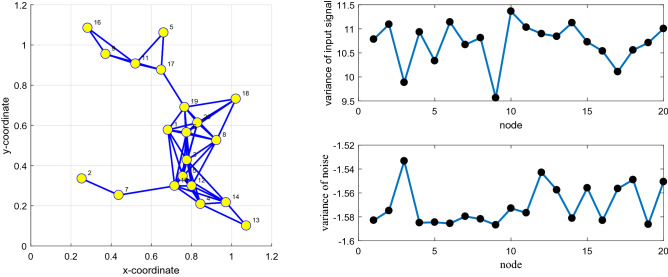
(Left) Random network topology to be decided by a certain radius; (Right_top) the input signals $\left\{ {{\mathbf{X}}_{n} \left( i \right)} \right\}$ variances of at each network node with ${\mathbf{R}}_{xx,n} = \sigma_{x,n}^{2} \left( t \right){\mathbf{I}}_{M} ,t = 1,2,3, \ldots ,M$, (Right_bottom) the measurement interference variances $\varepsilon_{n} \left( t \right)$ at each network node.

**Figure 12:**
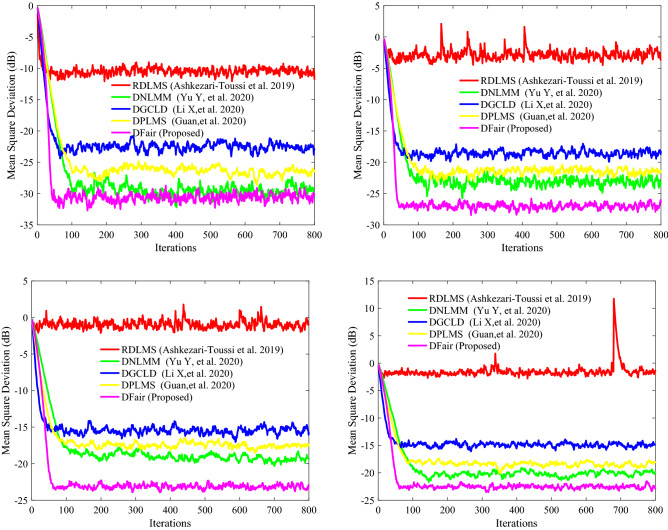
Transient network MSD (dB) iteration curves of the RDLMS, DNLMM, DGCLD, DPLMS, and DFair algorithms. (Up_Left) with ${\text{Pr}} = 0.1$, (Up_right) with ${\text{Pr}} = 0.2$, and (Down_left) with ${\text{Pr}} = 0.4$, and (Down_Right) with ${\text{Pr}} = 0.6$.

To illustrate our algorithm, it is more robust to ${\text{Pr }}$ for impulsive interference with a constant $\sigma_{f}^{2}$ and has a faster convergence rate and lower steady-state error than the RDLMS, DNLMM, DGCLD, and DPLMS algorithms. In this experiment, there have the same network topology, same $\sigma_{f}^{2} { }$ for impulsive interference and the same input signal. If any two nodes in network topology are declared neighbors, a certain radius for each node is larger than or equal to 0.3, and the network topology is shown in Fig. 11(Left). The MSD iteration curves for RDLMS ($\mu$ equal to 0.4), DNLMM ($\mu$ equal to 0.4), DGCLD ($\mu$ equal to 0.4), DPLMS, and DFair ($\mu$ equal to 0.4) algorithms in Fig. 12 with $\sigma_{f}^{2} = 0.04$ and the cut-value $\delta = 0.1$ for the DFair algorithm. Besides, the independent Monte Carlo number is 10, and each run has 800 iteration numbers.

In this experiment, four sub-experiments with different ${\text{Pr}}$ of impulsive interference were set with the same density of impulsive interference, the same input signal, and the same distributed network topology. From Fig. 12, we can find that although different ${\text{Pr}}$ of impulsive interference is considered, the DFair algorithm has a slightly faster rate than the RDLMS, DNLMM, DGCLD, and DPLMS algorithms. The DFair algorithm still has a minor steady-state error than the RDLMS, DNLMM, DGCLD, and DPLMS algorithms. In a word, from *Simulation experiment 3*, we can observe that the DFair algorithm is more robust to impulsive interference than the RDLMS, DNLMM, DGCLD, and DPLMS algorithms.

## Conclusion

This paper proposed a novel diffusion algorithm by using the Fair cost function, namely the DFair algorithm. The method is developed to combine and modify the DLMS algorithm and the Fair cost function at all distributed network nodes. Compared with some existing distributed adaptive filtering algorithms, the DFair algorithm has low computational complexity. The theoretical analysis demonstrates that the DFair algorithm can effectively estimate from an M-estimation cost function perspective. Besides, theoretical mean behavior interpreted that the DFair algorithm can achieve accurate estimation under the convergence interval. Besides, experimental simulation results showed that the DFair algorithm is more robust to the input signal and impulsive interference than the RDLMS, DNLMM, DGCLD, and DPLMS algorithms. Overall, the DFair algorithm has superior performance when estimating the unknown linear system under different input signals and the changeable impulsive interference environments.
